# Self-Generated Touch: A Neural Perspective

**DOI:** 10.1371/journal.pbio.0040048

**Published:** 2006-01-17

**Authors:** Liza Gross

Why can a person go into near hysterics when tickled by another, yet feel barely a trace of that sensation at the flutter of their own fingers? While there's little doubt that we perceive externally generated touch more acutely than we do our own, the underlying mechanisms for this attenuated response have remained unresolved. In a new study, Daniel Wolpert, J. Randall Flanagan, and Paul Bays present experimental evidence that offers new insight into this longstanding question.

Neuroscientists have long distinguished two possible mechanisms to explain this attenuated response. One mechanism relies on prediction: when a motor command is generated—touch face with right hand, for example—the brain predicts what the sensory consequence will be, based on previous experience. This predicted effect is removed from sensory signals sent to the brain, reducing the response. But attenuation could also result from processing that arises after the sensation is received, but before it is perceived. In such a nonpredictive, or postdictive, mechanism, sensory cues persist after the initial touch and undergo additional processing that is modified by inputs occurring around the same time.

Using a device outfitted with force sensors and a torque motor (to generate a stable force), the authors studied the neural mechanisms of self-generated touch in 20 volunteers. With their left hand palm-side up, participants rested their left index finger in a molded support, beneath a force-sensing device mounted on a lever attached to the torque motor. The apparatus allowed investigators to distort the force and sensations experienced when one finger touched the other. Specifically, subjects moved their finger to tap a sensor, which caused a motor to initiate taps to a second passive finger.

The authors designed three trials: contact, no contact, and a delay between the tap and the force generated. In a previous study, the authors had established that a 300-millisecond delay does not cause attenuation, so they used a 500-millisecond delay as a baseline measure. The perceived magnitude of taps during contact trials was, as the authors expected, significantly less than that reported for the baseline trials.

In no-contact trials—given just once out of every six trials—the top sensor was surreptitiously moved prior to a “go” signal (both sensors were hidden), so the person didn't touch the sensor. The test tap was triggered when the person's finger reached the flexion angle associated with contact. Again, the perceived magnitude of the test tap was “substantially reduced” compared with the delay trials. This result would be expected for a predictive mechanism: because the test taps in both contact and no-contact trials were given at the same time, a predictive mechanism would expect the sensation and so attenuate it. But a postdictive mechanism, which integrates sensory input from both fingers, would not recognize the test tap in no-contact trials as self-generated and, thus, not cause attenuation.

Still, it is possible, the authors explain, that a postdictive mechanism might rely on other cues, such as finger motion or position. To explore this possibility, they repeated their experiment with a second group of volunteers. But in these trials, the tapping finger never touched the sensor and the test tap was triggered as the tapping finger reached the position at which contact would have been made, or after a 500-millisecond delay. These participants perceived little difference between the two sets of trials, indicating that motion or location cues alone did not cause attenuation.

Since attenuation was perceived only for those participants given mostly contact trials whether or not contact occurred, these results argue for a predictive mechanism. What advantage could a predictive mechanism offer? It might allow us to rehearse movements in our mind before we carry them out, the authors suggest, compensating for irregularities in sensory processing to ensure an environmentally appropriate sensory response. And by heightening our sensitivity to external sensory cues, it may help focus our attention on those things more likely to affect our well being.

